# Factors associated with hand washing effectiveness: an institution-based observational study

**DOI:** 10.1186/s13756-023-01293-1

**Published:** 2023-08-30

**Authors:** Chen Shi, Margaret O’Donoghue, Lin Yang, Hilda Tsang, Jing Chen, Jing Zou, Jing Qin, Yim-Wah Mak, Didier Pittet, Yao Jie Xie, Timothy Lai, Chen Li, Jiannong Cao

**Affiliations:** 1 School of Nursing, The Hong Kong Polytechnic University, Hong Kong, China; 2 School of Public Health, Southern Medical University, Guangzhou, China; 3 Jockey Club School of Public Health and Primary Care, Faculty of Medicine, The Chinese University of Hong Kong, Hong Kong, China; 4 Faculty of Medicine, University of Geneva, Geneva, Switzerland; 5 Department of Computing, The Hong Kong Polytechnic University, Hong Kong, China; 6 Department of Applied Social Sciences, The Hong Kong Polytechnic University, Hong Kong, China

**Keywords:** Hand hygiene, Hand washing, Infection prevention and control, Standards

## Abstract

**Background:**

Few studies have investigated how the effectiveness of hand washing in removing hand contaminants is influenced by the performance and duration of each step involved. We conducted an observational study by recruiting participants from a university campus, with the aim to comprehensively evaluate how performance, duration and demographic factors influence hand washing effectiveness.

**Methods:**

A total of 744 videos were collected from 664 participants in July-October 2022 and independently evaluated by two infection control experts through labelling videos for correct and incorrect performance of each step. The individual hand washing effectiveness was determined by quantifying the percentage of residual fluorescent gel on the dorsum and palm areas of each participant’s hands. A logistic regression analysis was conducted to identify factors that were significantly associated with better hand washing effectiveness. An exposure-response relationship was constructed to identify optimal durations for each step. Approximately 2300 hand images were processed using advanced normalization algorithms and overlaid to visualize the areas with more fluorescence residuals after hand washing.

**Results:**

Step 3 (rub between fingers) was the most frequently omitted step and step 4 (rub the dorsum of fingers) was the most frequently incorrectly performed step. After adjustment for covariates, sex, performance of step 4 and step 7 (rub wrists), rubbing hands during rinsing, and rinsing time were significantly associated with hand washing effectiveness. The optimal overall hand washing time was 31 s from step 1 to step 7, and 28 s from step 1 to step 6, with each step ideally lasting 4–5 s, except step 3. The palms of both hands had less fluorescence residuals than the dorsums. The areas where residuals most likely appeared were wrists, followed by finger tips, finger webs and thumbs.

**Conclusions:**

Performance and duration of some hand washing steps, sex and rinsing time were associated with hand washing effectiveness. The optimal duration might be applied to all seven steps to achieve the best decontamination results. Further studies are needed to refine hand hygiene standards and enhance compliance.

**Supplementary Information:**

The online version contains supplementary material available at 10.1186/s13756-023-01293-1.

## Background

Healthcare associated infections (HAI) have been associated with longer hospital stay, increased mortality and morbidity in patients, and cause high economic burden globally. The overall prevalence rate of HAI was estimated to be 5.7% and 9.0% in Europe and Southeast Asia, respectively [1, 2]. Zimlichman et al. estimated HAIs caused an annual economic cost of $9.8 billion in the United States [3]. There is ample evidence to demonstrate the effectiveness of hand hygiene on reducing HAI and spreading of multi-drug resistant organisms (MRDO) [4]. For example, a hospital-wide hand hygiene campaign was found to reduce overall HAI prevalence and methicillin-resistant staphylococcus aureus (MRSA) transmission rates by 40% and 57%, respectively [5]. Therefore, hand hygiene is one of the core components of infection prevention and control (IPC) programs in healthcare settings [6].

As part of IPC auditing programs in healthcare settings, current surveillance programs for hand hygiene compliance in healthcare settings mainly focus on five moments for hand hygiene, but few attention is paid upon the results of individual hand hygiene performance in clinical practice [7]. The World Health Organization (WHO) guidelines on hand hygiene in health care recommends six-step techniques for effective hand hygiene: (1) rub both hands palm to palm; (2) rub the dorsum of each hand with the palm of the other hand, with fingers interlaced; (3) rub palm to palm, with fingers interlaced; (4) rub the dorsums of fingers against the opposite palm, with fingers interlocked; (5) rub one thumb by palm of the other hand, and rub the other thumb; (6) rub the tips of your fingers [8]. Previous laboratory studies found that the six-step hand hygiene procedure should take 20–30 s if using alcohol-based hand rub (ABHR), or 40–60 s if washing hands with soap and water, in order to ensure effective removal of transient microorganisms acquired from direct contact with patients, contaminated surfaces, or the environment [8]. Some health authorities, including the Center for Health Protection (CHP) in Hong Kong, also recommends one extra step of rubbing wrists so that there are a total of seven steps [9]. Most previous studies focused on the total duration of hand hygiene [7, 10].

Prevous studies have reported a few factors associated with hand washing effectiveness. For example, rubbing hands under running added additional physical friction which improved the decontamination outcome of hands [11]. An institution-based study found that medical students had better knowledge and performance of hand washing compared to the students from other disciplines [12]. A few studies also found age and gender differences in knowledge, practive and effectivenss hand hygiene [13, 14]. However, no studies have evaluated the duration needed for each step and the factors influencing the cleaning effectiveness of hand hygiene.

In the study, we aimed to comprehensively evaluate the factors associated with hand wash effectiveness, using video recordings and a ultraviolet (UV) hand scanner to objectively measure residual florescent contaminants after hand washing.

## Methods

### Subject recruitment and data collection

A convenient sampling method was used to recruit participants from the Hong Kong Polytechnic University campus using posters during 8 July – 14 October 2022. People with clinical experiences or hands-on trainings of hand washing were excluded. After signing a consent form, participants were asked to provide demographic information such as age, sex, staff or students, and departments/programs.

Before washing hands, participants were requested to put fluorescent lotion (GlitterBug™, Brevis Corporation, the United States) on both of their hands and scan their hands using the hand scanner (Semmelweis Scanner™, RDI Systems Ltd, Ireland) to ensure that sufficient powder covered all hand areas including both wrists. Participants then performed seven steps of hand wash without onsite training nor receiving instruction from researchers. The accuracy of this hand scanner in hand washing assessments has been validated by comparing with traditional testing methods such as bacteria agar culture [15]. The camera installed above water basins started to record both hands when research assistants turned on water faucets, and stopped after participants rinse their hands with water. After washing hands, participants scanned their both hands using the hand scanner again, to record the percentages of hand areas with residual fluorescent gel of palm and dorsum of left and right hands after hand washing, respectively. A few participants had more than one attempts of hand washing which were separately labelled and analyzed.

Two IPC experts independently judged the correctness of hand washing steps by watching recorded hand washing videos of all participants. They labeled the videos by fragments according to seven steps, and corresponding start and end time points of each fragment were marked to calculate total duration time. The correctness of hand washing performance for each video fragment was further scored with 0 for missing step, 1 for correct step, 2 for wrong step, and 3 for no-label due to blocked views. We further classified individual hand washing effectiveness for each step into four types by combining all video fragment scores related to this step: 1) completely correct (all fragments were labeled with 1; 2) partially correct (some videos were labeled with 1 and the rest were 2); 3) completely wrong (all fragments were labeled with 2); 4) missing (all fragments were labeled with 0). Total duration of each step (at the smallest unit of one second), rinsing time, and rubbing hands when rinsing were also recorded when labelling videos.

### Visualization of hand areas with contaminant residuals

We visualized common areas of left and right hand palm and dorsum, by combining all hand images taken by the hand scanner after hand washing. The images were first converted to grayscale by removing unrelated color information and focusing on intrinsic characteristics of these images. We randomly selected the images of one participant as templates, and the rest images were subsequently registered to the corresponding template one by one, using the advanced normalization tools [16]. This image registration process consisted of two steps: affine registration and nonlinear registration. Affine registration was to achieve a preliminary alignment of the input images with the template images, by applying translation, rotation, scaling, and shearing transformations. The subsequent nonlinear registration employed a deformable registration approach to achieve more precise and elaborate registration. Specifically, the symmetric normalization (SyN) algorithm [17], which incorporates both forward and backward mappings for bidirectional registration and alignment, was utilized to capture deformations present in the hand images. The nonlinear registration process addressed variations in hand shape, size, and local deformations, resulting in a highly accurate alignment of the input images to the template images.

All registered images were then normalized to a common intensity range of 0 (no fluorescence residual) to 1 (maximum fluorescence residual among all participants), and finally overlaid in a color map to visualize the areas with fluorescence residuals.

### Statistical analysis

We defined the outcome measurement as classification of individual participants into the good and poor hand washing groups by the maximum of the percentages of hand areas of palm or dorsum of left and right hands with residual fluorescent gel after hand washing. Specifically, we adopted the cut-off of 1%, which approximated the median values of maximum residual percentage of individual participants. That is to say, if one participant had > 1% of his/her hand areas of palm or dorsum of left and right hands with residual fluorescent gel after hand washing, he/she would be classified into the poor hand washing performance group.

We explored potential influencing factors related to hand washing effectiveness by fitting univariate logistic regression models with only one variable in each model. The variables included demographics, video label of each step, duration of performing correct, wrong hand washing steps, rubbing hands when rinsing, duration of rinsing hands, and total duration time of all steps. Two multivariate logistic regression models were fitted to the data, respectively. The first was a full model which included all variables that were statistically significant (*p* < 0.05) in univariate logistic regression analyses. The generalized variance inflation factor (GVIF) was calculated for each candidate variable to evaluate the potential multicollinearity [18]. The second model was a simplified model by selecting variables with backward selection and minimal Akaike information criterion (AIC). Odds ratio (OR) was derived from the logistic regression models to estimate the effect of individual factors.

The exposure-response curve between duration of each step and hand washing performance was subsequently constructed by adding a restricted cubic splines function to the multivariate logistic regression model that also included sex, department and any other potential independent factors.

We conducted a sensitivity analysis by removing all outliers as revealed in the box plots of duration of each step, and repeated all the above statistical analyses. To show the robustness of our findings against the cut-off values in defining the good and poor hand washing groups as outcome, we also conducted sensitivity analyses by changing the cut-off values to the median of maximum and average residual percentages of individual participants (0.835% and 0.37%), respectively. In this study, p < 0.05 was considered statistically significant. R software V.4.1.1 (R Foundation for Statistical Computing) was used for all analyses.

The study was approved by the Hong Kong Polytechnic University Institutional Review Board (Reference Number: HSEARS20220519005).

## Results

**Fig. 1:**
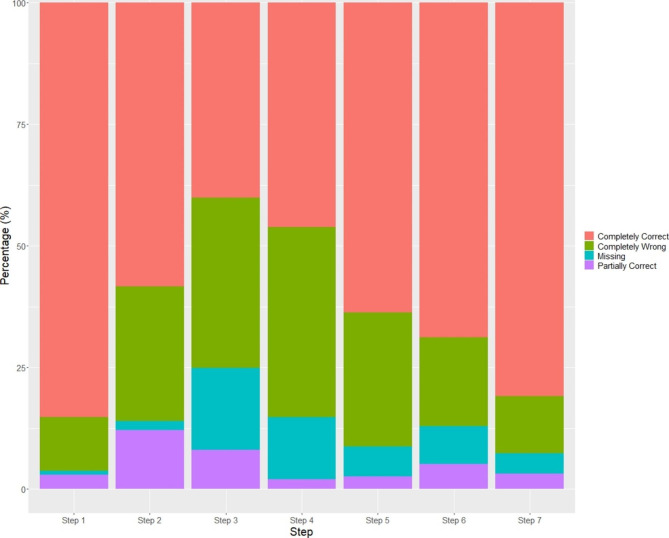
Percentage of participants in different performance categories by seven steps

We collected 744 videos from 664 participants. The age of these participants ranged from 20 to 22 years (median 21.0 years). The majority were females (537, 72.9%) and nursing students (658, 92%) (Table 1). The non-nursing participants included students and staff from business, sciences, engineering, social science and supporting departments. 399 videos (53.6%) were defined as good hand wasing effectiveness (residual percentage ranged from 0.03 to 1%), and 345 videos (46.4%) as poor (residual percentage ranged from 1.01 to 97.2%). 14% of the participants performed all seven steps correctly and met the minimal duration of 20 s for hand rubbing. None of them completely removed fluorescent contaminants (i.e. zero residual after washing). Most people (85.2%) performed step 1 (rub palm to palm) completely correctly, whereas step 3 (rub between fingers) was the most frequently (16.8%) ignored step (Fig. 1). There were 39.1% of the participants who performed step 4 (rub dorsum of fingers) in a completely wrong way, and 12.1% performed step 2 (dorsum of each hand) partially correctly.

**Table 1:** Comparison of characteristics and variables between the participants with good and poor hand washing effectiveness

Variables	All	Good effectiveness	Poor effectiveness	Odds Ratio
	(n = 744)	(n = 399)	(n = 345)	(95%CI)
Age, years, median (IQR)	21 (20–22)	21 (20–22)	21 (20–22)	1.00 (0.98–1.03)
Sex, female, n (%)	537/737 (72.9)	327/397 (82.4)	210/340 (61.8)	2.89 (2.07–4.07)
Nursing students, n (%)	658/715 (92.0)	368/391 (94.1)	290/324 (89.5)	1.88 (1.09–3.29)
Step 1, n (%)				
Missing	6/744 (0.8)	3/399 (0.8)	3/345 (0.9)	Reference
Completely correct	634/744 (85.2)	339/399 (85.0)	295/345 (85.5)	1.15 (0.21–6.25)
Partially correct	22/744 (3.0)	13/399 (3.3)	9/345 (2.6)	1.44 (0.22–9.46)
Completely wrong	82/744 (11.0)	44/399 (11.0)	38/345 (11.0)	1.16 (0.20–6.58)
Step 2, n (%)				
Missing	14/744 (1.9)	5/399 (1.3)	9/345 (2.6)	Reference
Completely correct	434/744 (58.3)	242/399 (60.7)	192/345 (55.7)	2.27 (0.77–7.49)
Partially correct	90/744 (12.1)	48/399 (12.0)	42/345 (12.2)	2.06 (0.66–7.14)
Completely wrong	206/744 (27.7)	104/399 (26.1)	102/345 (29.6)	1.84 (0.61–6.15)
Step 3, n (%)				
Missing	125/744 (16.8)	60/399 (15.0)	65/345 (18.8)	Reference
Completely correct	298/744 (40.1)	156/399 (39.1)	142/345 (41.2)	1.19 (0.78–1.81)
Partially correct	60/744 (8.1)	34/399 (8.5)	26/345 (7.5)	1.42 (0.76–2.65)
Completely wrong	261/744 (35.1)	149/399 (37.3)	112/345 (32.5)	1.44 (0.94–2.21)
Step 4, n (%)				
Missing	95/744 (12.8)	45/399 (11.3)	50/345 (14.5)	Reference
Completely correct	343/744 (46.1)	192/399 (48.1)	151/345 (43.8)	1.41 (0.90–2.23)
Partially correct	15/744 (2.0)	12/399 (3.0)	3/345 (0.9)	4.44 (1.31–20.42)
Completely wrong	291/744 (39.1)	150/399 (37.6)	141/345 (40.9)	1.18 (0.74–1.88)
Step 5, n (%)				
Missing	46/744 (6.2)	17/399 (4.3)	29/345 (8.4)	Reference
Completely correct	474/744 (63.7)	262/399 (65.7)	212/345 (61.4)	2.11 (1.14–4.01)
Partially correct	19/744 (2.6)	10/399 (2.5)	9/345 (2.6)	1.90 (0.64–5.70)
Completely wrong	205/744 (27.6)	110/399 (27.6)	95/345 (27.5)	1.98 (1.03–3.88)
Step 6, n (%)				
Missing	58/744 (7.8)	25/399 (6.3)	33/345 (9.6)	Reference
Completely correct	512/744 (68.8)	277/399 (69.4)	235/345 (68.1)	1.56 (0.90–2.71)
Partially correct	38/744 (5.1)	27/399 (6.8)	11/345 (3.2)	3.24 (1.38–7.99)
Completely wrong	136/744 (18.3)	70/399 (17.5)	66/345 (19.1)	1.40 (0.76–2.62)
Step 7, n (%)				
Missing	31/744 (4.2)	9/399 (2.3)	22/345 (6.4)	Reference
Completely correct	602/744 (80.9)	328/399 (82.2)	274/345 (79.4)	2.93 (1.37–6.80)
Partially correct	24/744 (3.2)	17/399 (4.3)	7/345 (2.0)	5.94 (1.91–20.35)
Completely wrong	87/744 (11.7)	45/399 (11.3)	42/345 (12.2)	2.62 (1.11–6.60)
Correct duration, seconds, median (IQR)				
Step 1	3 (2–5)	3 (2–5)	3 (2–5)	0.95 (0.91–0.99)
Step 2	4 (0–8)	4 (0–7)	4 (0–8)	0.99 (0.96–1.01)
Step 3	0 (0–3)	0 (0–3)	0 (0–4)	0.96 (0.91–1.00)
Step 4	0 (0–6)	2 (0–6)	0 (0–6)	1.00 (0.97–1.03)
Step 5	4 (0–7)	4 (0–7)	4 (0–7)	1.00 (0.97–1.03)
Step 6	4 (0–7)	4 (1–7)	4 (0–7)	0.99 (0.96–1.01)
Step 7	5 (3–8)	5 (3–8)	5 (3–8)	0.99 (0.96–1.02)
Partially correct/completely wrong duration, seconds, median (IQR) Step 1	0 (0–0)	0 (0–0)	0 (0–0)	1.01 (0.90–1.13)
Step 2	0 (0–3)	0 (0–3)	0 (0–3)	0.98 (0.95–1.01)
Step 3	0 (0–2)	0 (0–2)	0 (0–2)	1.01 (0.96–1.06)
Step 4	0 (0–3)	0 (0–3)	0 (0–4)	0.98 (0.94–1.02)
Step 5	0 (0–1)	0 (0–1)	0 (0–1)	1.00 (0.95–1.05)
Step 6	0 (0–0)	0 (0–0)	0 (0–0)	1.00 (0.96–1.05)
Step 7	0 (0–0)	0 (0–0)	0 (0–0)	1.01 (0.91–1.11)
Rubbing when rinsing hands, n (%)	646/744 (86.8)	361/399 (90.5)	285/345 (82.6)	2.00 (1.30–3.11)
Rinsing time	17 (13-24.25)	18 (13–25)	16 (12–23)	1.02 (1.01–1.04)
One hand washing attempt, n (%)	658/744 (88.4)	379/399 (95.0)	279/345 (80.9)	0.22 (0.13–0.37)
Total duration of step 1–7 regardless of correctness	35 (23–51)	34 (23–50)	36 (23–53)	1.00 (0.99–1.00)
Total duration of correct steps 1–7	27 (14–41)	26 (14–39)	27 (14–44)	1.00 (0.99–1.00)
Total duration of step 1–6 regardless of correctness	29 (19–43)	28 (19–42)	31 (19–45)	0.99 (0.99–1.00)
Total duration of correct steps 1–6	21 (11–32)	20 (11–31)	22 (10–35)	0.99 (0.99–1.00)

In univariate analysis, females, nursing students, rubbing hands when rinsing with water, total rinsing time, and one hand washing attempt were significantly associated with better hand washing results (Table 1). Performance of step 4 to 7 and correct performance duration of step 1 were associated with hand washing effectiveness. No statistically significant differences were found in total duration of hand washing (either including or excluding incorrect performance duration) between the participants with good or poor hand washing effectiveness (Table 1).

The multivariate model included the variables of sex, nursing students, correctness of step 4, 5, 6 and 7, duration of step 1, rubbing hands when rinsing, and only one attempt of hand washing (Table 2). None of these variables was excluded after checking multicollinearity in this model (Addtional file 1). After backward selection, the effect estimates from the simplified model were significant for female (vs. male OR) = 2.60, 95% CI 1.80 to 3.78), step 4 performance (partially correctly vs. missing OR = 6.23, 95% CI 1.55 to 32.99), step 7 performance (completely correctly vs. missing OR = 2.77, 95% CI 1.12 to 7.23, or partially correctly vs. missing OR = 5.89, 95% CI 1.65 to 23.29), rubbing hands when rinsing (OR = 2.00, 95% CI 1.25 to 3.23), and rinsing duration (OR = 1.02, 95% CI 1.01 to 1.04). More than one hand washing attempts (OR = 0.25, 95% CI 0.14 to 0.46) and longer step 1 duration (OR = 0.95, 95% CI 0.90 to 1.00) were significantly associated with poor hand washing effectiveness (Table 2).

**Table 2:** Results of multivariate logistic regression models

Characteristics	Full model			Simplified model	
	OR (95%CI)	P value	OR (95%CI)		P value
Male	Reference		Reference		
Female	2.70 (1.86–3.95)	< 0.001	2.60 (1.80–3.78)		< 0.001
Others	Reference				
Nursing students	1.32 (0.61–2.82)	0.476	-		-
Step 4					
Missing	Reference	-	Reference		-
Completely correct	1.27 (0.68–2.35)	0.449	1.41 (0.80–2.47)		0.233
Partially correct	5.65 (1.34–31.03)	0.027	6.23 (1.55–32.99)		0.017
Completely wrong	1.00 (0.54–1.81)	0.990	1.15 (0.66–1.99)		0.623
Step 5					
Missing	Reference	-	-		-
Completely correct	1.57 (0.63–3.93)	0.330	-		-
Partially correct	1.73 (0.45–6.99)	0.433	-		-
Completely wrong	1.41 (0.58–3.49)	0.450	-		-
Step 6					
Missing	Reference	-	-		-
Completely correct	0.75 (0.34–1.66)	0.485	-		-
Partially correct	1.58 (0.55–4.63)	0.399	-		-
Completely wrong	0.69 (0.30–1.56)	0.382	-		-
Step 7					
Missing	Reference	-	Reference		-
Completely correct	2.35 (0.77–7.38)	0.134	2.77 (1.12–7.23)		0.031
Partially correct	4.29 (1.05–18.8)	0.046	5.89 (1.65–23.29)		0.008
Completely wrong	2.23 (0.72–7.08)	0.165	2.42 (0.93–6.66)		0.076
Step 1 correct duration	0.94 (0.90–0.99)	0.030	0.95 (0.90–1.00)		0.039
Rubbing hands when rinsing	2.04 (1.27–3.31)	0.003	2.00 (1.25–3.23)		0.004
Rinsing duration	1.02 (1.01–1.04)	0.002	1.02 (1.01–1.04)		0.002
One hand washing attempt	0.27 (0.14–0.48)	< 0.001	0.25 (0.14–0.46)		< 0.001

**Fig. 2:**
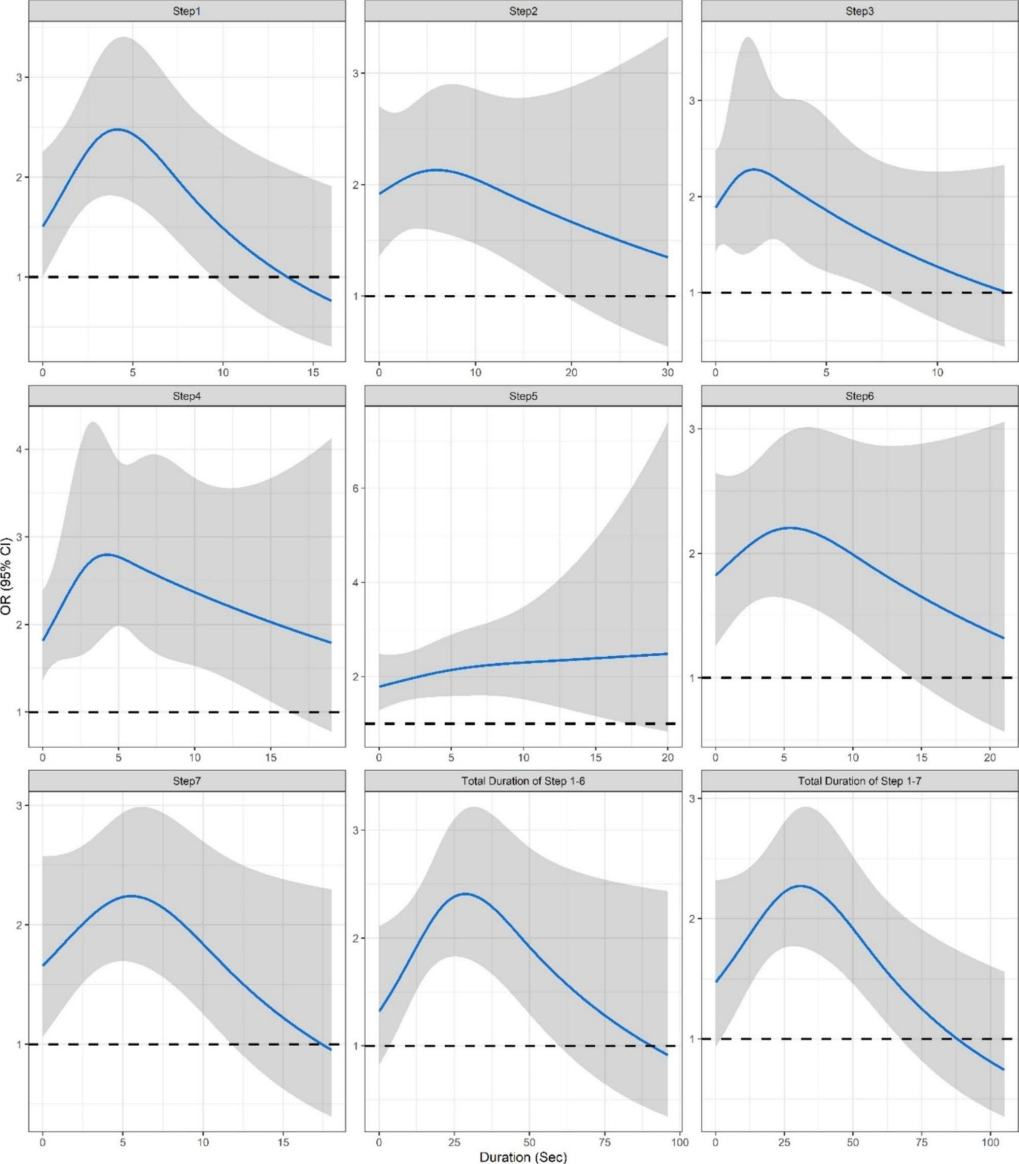
Exposure-response curve on hand washing effectiveness by total duration and duration of each step. The blue lines are odd ratio point estimates and grey bands highlight the 95% confidence interval

Figure 2 shows the exposure-response curve of total duration of correct hand washing and duration of correct performance of each step on hand wash effectiveness, after adjustment for sex, age, department, number of attempts, rubbing hands when rinsing, rinsing time and duration of other steps. The duration that was associated with the best effect (i.e. highest OR) was two seconds for step 3, four seconds for step 1 and step 4, five seconds for step 6 and step 7, six seconds for step 2, respectively. The curve of step 5 reached a plateau beyond five seconds. The effect estimates associated with total duration of step 1 to step 6 peaked at 28 s, and 31 s if step 7 is included. The results of sensitivity analyses by removing outliers were consistent with main findings (Additional file 2).

**Fig. 3:**
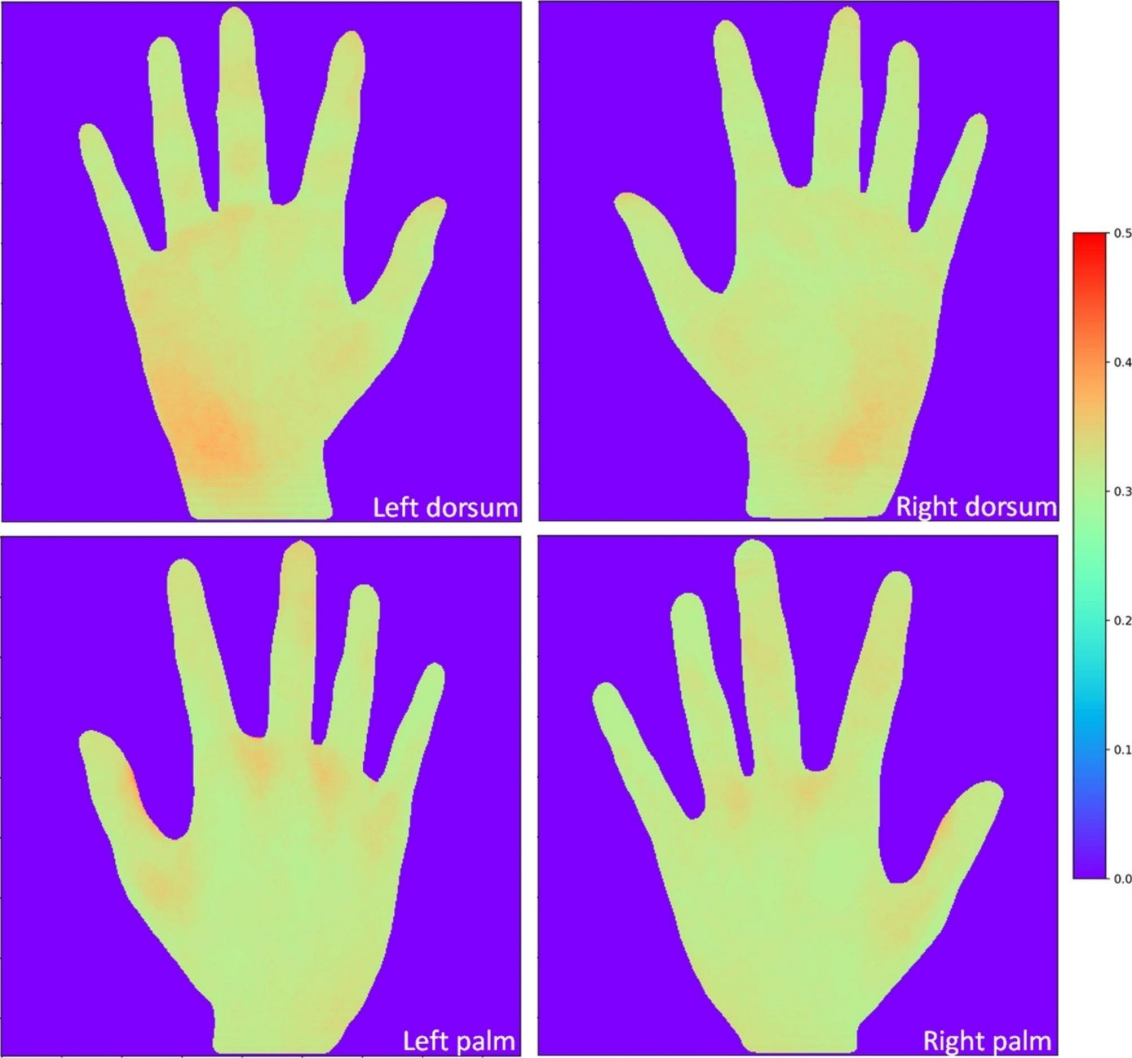
Overlaid color map images of palm and dorsum of left/right hand after normalization. The color ranges from 0 (no fluorescent residual) to 1 (maximum fluorescent residual)

Approximately 2300 hand images (four images from each participant) were processed and registered using the advanced normalization algorithms. All the normalized images were overlaid for the palm and dorsum of left/right hands, respectively (Fig. 3). The palms of both hands had less fluorescence residuals than the dorsum. The areas where residuals most likely appeared were wrists, followed by finger tips, finger webs and thumbs.

## Discussion

In this study, we found that female, correct performance of steps 4 and 7, shorter duration of step 1, longer rinsing time, and rubbing hands when rinsing were significantly associated with better hand washing results (less residual contaminants after washing). To our best knowledge, this is the first study to conduct an in-depth analysis on a large sample of video recordings and hand images, with the aim to explore the influencing factors of hand washing effectiveness by seven step.

The use of fluorescent gel in hand washing was found to be an effective and novel approach to improve hand hygiene education. Vanyolos et al. and Szilágyi et al. used fluorescent gel with UV light to assess quality of handwashing and found that females wash their hands better than males, and persons with nurse background had the best handwashing practices [19, 20]. Our results showed that wrists and fingertips had the most residuals after hand washing. Similar findings were reported by Szilágyi et al. who found that the most common missed area were the wrists and fingernails, and by a local study identified fingertips as the most missed areas [20, 21]. The results in this study were comparable to other studies using similar fluorescent gel assessment technique. However, there were no previous studies that investigated the impact of step-by-step hand hygiene durations on effective removal of hand contaminants as we did in this study.

One of the unresolved issues in hand hygiene studies is lack of strong evidence to support the current recommendations on duration of hand hygiene [22]. The WHO recommends that the duration of the entire procedure for hand washing lasts 40–60 s (from wet hands with water to dry hands), but not giving detailed instructions on duration of each step [7]. It is of note that such recommendations were supported by a few experimental studies on hand wash effectiveness in reducing (but not eliminating) certain bacteria [23, 24]. We used regression models to assess the dose-response relationship with adjustment of other covariates, and found that the optimal duration was 31 s for all seven steps and 28 s for the first six steps combined and ranged from two to six seconds for each step. A review by the United States Centers for Disease Control and Prevention (USCDC) found that the average duration of hand washing episodes ranged from 6.6 to 24.0 s in previous observational studies [25], which was also shorter than the optimal duration found in our study. It is of note that 15–20 s of minimal scrubbing time are recommended by most hand hygiene guidelines for ABHR, including the USCDC [26], the Society for Healthcare Epidemiology (SHEA) [27], the European Center for Disease Prevention and Control (ECDC) [28], and the CHP in Hong Kong [9]. However, few of these guidelines provided strong evidence to support these recommendation. Future studies are needed to investigate the optimal duration of hand washing and ABHR to achieve the highest effectiveness in removing hand contaminants in real clinical settings.

In our study, only 14.4% of participants performed seven steps all correctly. Although the majority have received prior trainings in lectures or tutorials, none have previously taken individual hands-on trainings for hand washing. This reflects trainings via lectures or group tutorials may not be sufficient for students to comprehend hand washing techniques. We also found that attempting more than once was statistically significantly associated with poor hand washing effectiveness. This reveals it is difficult to master hand washing steps in a short time. More targeted training should be implemented to achieve effective hand washing [29].

Few hand hygiene guidelines have taken into consideration of wrist areas. Although wrists are less likely to directly contact with surfaces, patients, and medical equipment, as compared to palm, dorsum of hands and fingers, the risk of contamination and spreading bacteria by wrists of healthcare workers cannot be completely ignored.

A meta-analysis reported the weighted pooled compliance rate for nurses was 52% and for doctors was 45% [30], which were lower than 60%, the optimal threshold of hand hygiene compliance rate for healthcare workers associated with the lowest hospital-acquired infections incidence rate [31]. Further studies may provide more information as to whether the standards of hand hygiene should be fine-tuned to improve hand hygiene compliance. For example, give a clearer requirement for rinsing time and duration of each step. As time pressure could be a major barrier to compliance, this could have a positive influence on the frequency of hand hygiene [32].

Our study has several potential caveats. First, most participants were nursing students from one university, who had attended lectures on hand hygiene but never received any hand-on trainings nor had any clinical experiences before joining the study. Hence our findings might not be generalizable to other populations such as healthcare professionals and the general public. Second, we used UV light, in combination with a florescent lotion or gel that is applied to the hands before washing, to mimic residual bacteria or other contaminants after washing. However, fluorescent lotion contaminants may not reflect the true effectiveness in clinical practices and more studies from other populations are needed to confirm our research findings. In future, we may consider using bacteria culture as endpoints to confirm our findings. Nevertheless, bacteria culture requires extra costs and manpower, therefore fluorescent lotion and hand scanners have become more commonly adopted in healthcare settings for routine monitoring of hand hygiene practices.

## Conclusions

Our study is among the first to explore the optimal duration of each step to maximize hand washing effectiveness. Performance and duration of some hand washing steps, sex and rinsing time were associated with hand washing effectiveness. Further studies are needed to refine hand hygiene standards and enhance compliance.

### Electronic supplementary material

Below is the link to the electronic supplementary material.

**Additional file 1: Table A1**. Generalized variance inflation factors for factors associated with hand washing effectiveness.

**Additional file 2: Fig. A1:** Exposure-response curve on hand washing effectiveness by total duration and duration of each step when outliers were removed. The blue lines are odd ratio point estimates and grey bands highlight the 95% confidence interval.

## Data Availability

The datasets used and analyzed during the current study are available from the corresponding author on reasonable request.

## Abbreviations

95% CI: 95% confidence interval
ABHR: alcohol-based hand rub
AIC: Akaike information criterion
ECDC: European Center for Disease Prevention and Control
CHP: Center for Health Protection
GVIF: generalized variance inflation factor
HAI: healthcare associated infections
IPC: infection prevention and control
MRDO: multi-drug resistant organisms
MRSA: methicillin-resistant staphylococcus aureus
OR: odds ration
SHEA: Society for Healthcare Epidemiology
SyN: symmetric normalization
USCDC: Centers for Disease Control and Prevention, United States
UV: ultraviolet
WHO: World Health Organization
