# Correction: Factors associated with hand washing effectiveness: an institution-based observational study

**DOI:** 10.1186/s13756-023-01313-0

**Published:** 2023-09-29

**Authors:** Chen Shi, Margaret O’Donoghue, Lin Yang, Hilda Tsang, Jing Chen, Jing Zou, Jing Qin, Yim-Wah Mak, Didier Pittet, Yao Jie Xie, Timothy Lai, Chen Li, Jiannong Cao

**Affiliations:** 1 School of Nursing, The Hong Kong Polytechnic University, Hong Kong, China; 2 School of Public Health, Southern Medical University, Guangzhou, China; 3 Jockey Club School of Public Health and Primary Care, Faculty of Medicine, The Chinese University of Hong Kong, Hong Kong, China; 4 Faculty of Medicine, University of Geneva, Geneva, Switzerland; 5 Department of Computing, The Hong Kong Polytechnic University, Hong Kong, China; 6 Department of Applied Social Sciences, The Hong Kong Polytechnic University, Hong Kong, China

**Antimicrobial Resistance & Infection Control (2023) 12:85**

10.1186/s13756-023-01293-1

The original article [1] contains a typo in the following sentence in the Methods section:

“[…] hands using the hand scanner (Semmelweis Scanner™, RDI Systems Ltd, Ireland)”

The correct sentence should instead be as follows:

“[…] hands using the hand scanner (Semmelweis Scanner™, HandInScan Zrt., Debrecen, Hungary)”

