# Supplementary material for: Factors associated with hand washing effectiveness: an institution-based observational study
Source: Antimicrob Resist Infect Control. 2023 Aug 30;12:85. doi: 10.1186/s13756-023-01293-1 (PMC10469426; doi:10.1186/s13756-023-01293-1)
Supplement: Supplementary file 1 — Additional file 1: Table A1. Generalized variance inflation factors for factors associated with hand washing effectiveness. [file 13756_2023_1293_MOESM1_ESM.pdf]

Additional Table A. Generalized variance inflation factors for factors associated with hand washing effectiveness

Variables	GVIF	*df*	GVIF $^{(1/(2\times df))}$
Female vs male	1.050	1	1.025
Nursing students vs others	1.563	1	1.250
Score for step 4	1.647	3	1.087
Score for step 5	2.032	3	1.125
Score for step 6	2.090	3	1.131
Score for step 7	2.171	3	1.138
Step 1 correct duration	1.168	1	1.081
Rubbing hands when rinsing	1.053	1	1.026
Rinsing duration	1.038	1	1.019
One attempt vs more than one attempts	1.120	1	1.058

Abbreviations: GVIF, generalized variance inflation factor; *df*, degree of freedom
