# Supplementary material for: Factors associated with hand washing effectiveness: an institution-based observational study
Source: Antimicrob Resist Infect Control. 2023 Aug 30;12:85. doi: 10.1186/s13756-023-01293-1 (PMC10469426; doi:10.1186/s13756-023-01293-1)

Additional Figure A. Exposure-response curve on hand washing effectiveness by total duration and duration of each step. The blue lines are odd ratio point estimates and grey bands highlight the 95% confidence interval.

A) When outliers were removed.

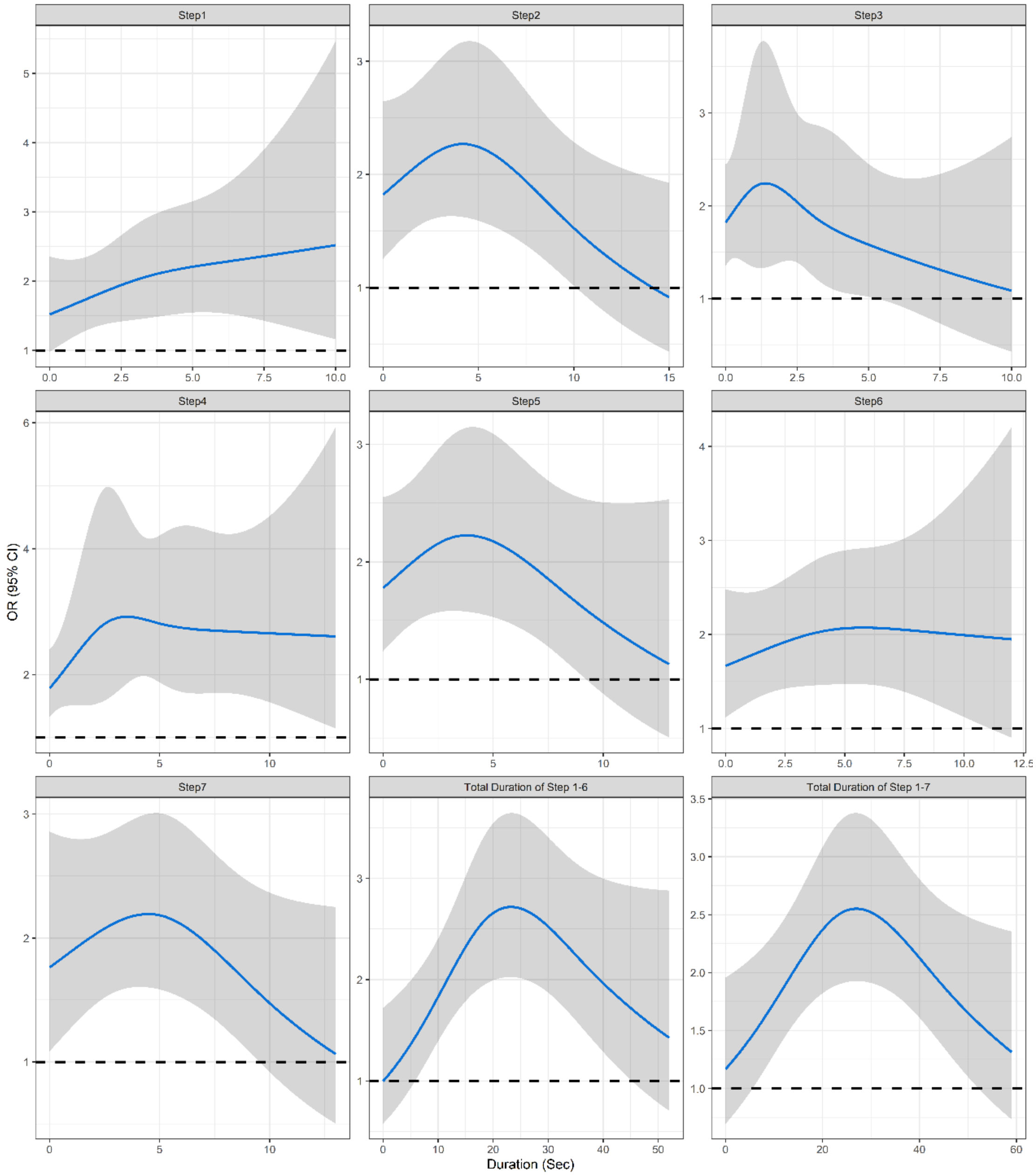

B) When cut-off for hand washing performance was 0.835% (median percentage).

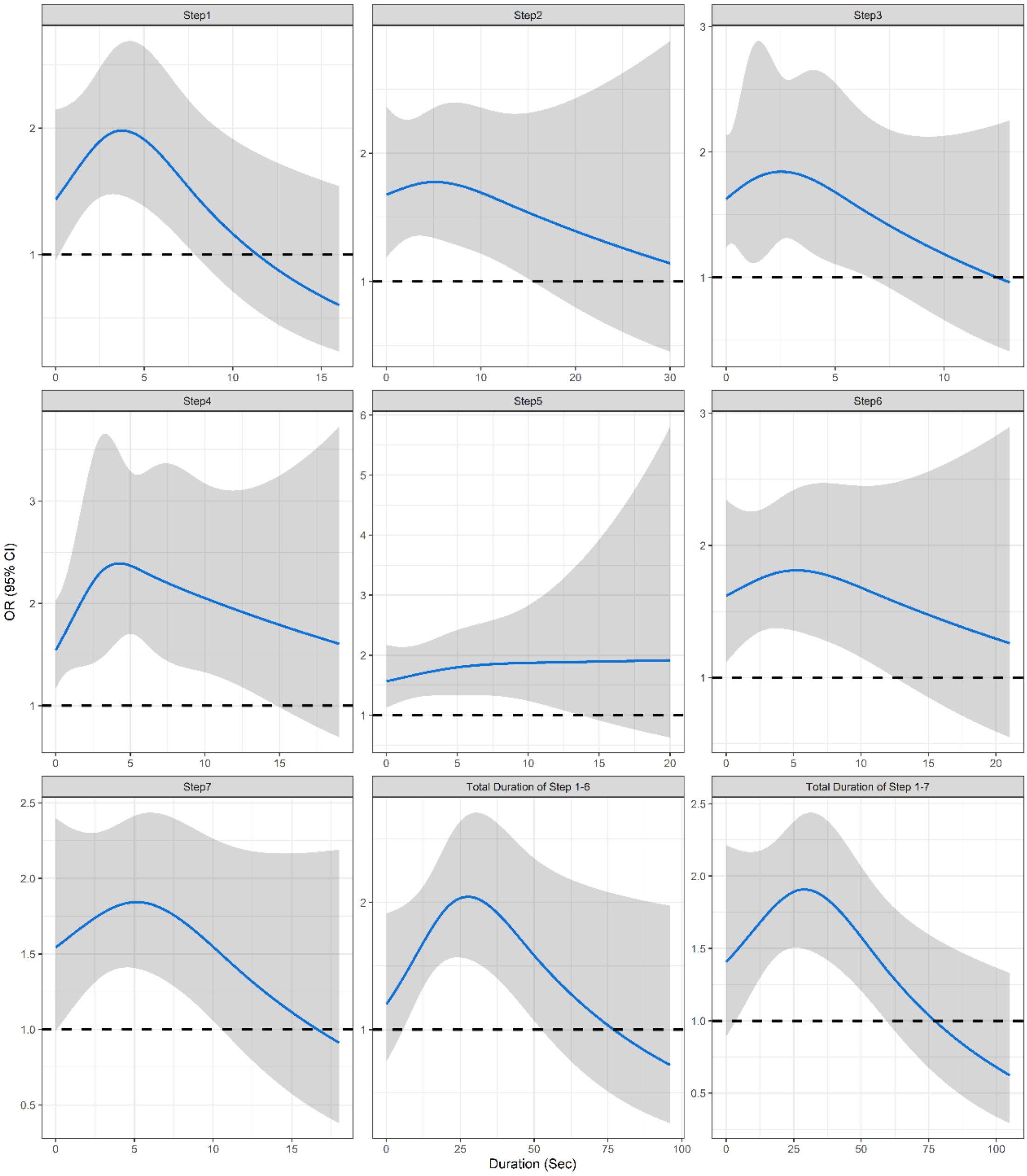

C) Calculating the arithmetic mean of the percentages of the four hand areas with residual fluorescent gel after hand washing to measure individual hand washing effectiveness and using 0.37% (median percentage) as the cut-off.

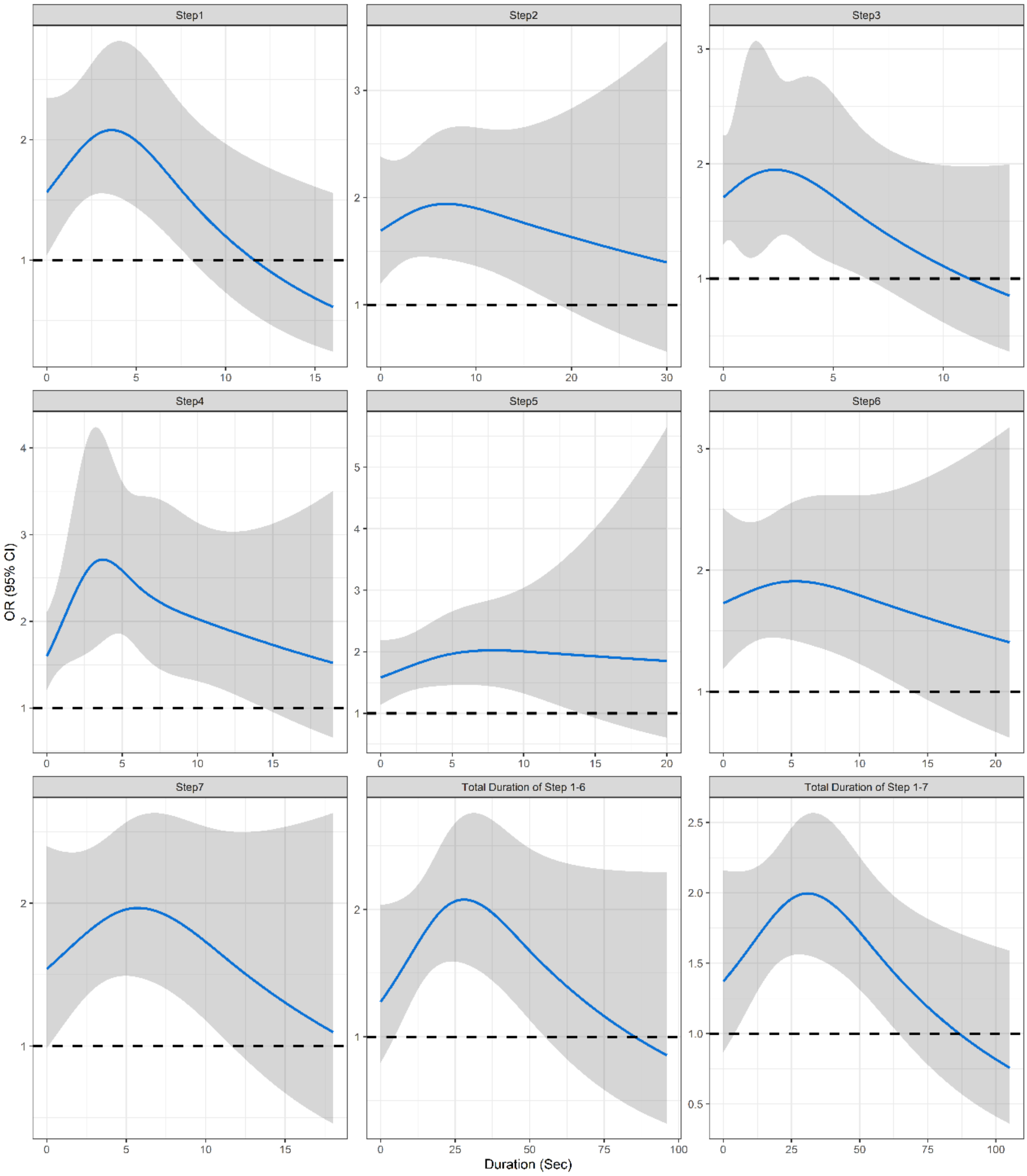

Supplement: Supplementary file 2 — Additional file 2: Fig. A1: Exposure-response curve on hand washing effectiveness by total duration and duration of each step when outliers were removed. The blue lines are odd ratio point estimates and grey bands highlight the 95% confidence interval. [file 13756_2023_1293_MOESM2_ESM.pdf]
